# Overlap and Divergence in Ketamine and Lithium Response in Bipolar Disorder: A Scoping Review

**DOI:** 10.3390/ph18111662

**Published:** 2025-11-03

**Authors:** Jay Toulany, Jasmyn E. A. Cunningham, Abraham Nunes

**Affiliations:** 1 Department of Psychiatry, Faculty of Medicine, Dalhousie University, Halifax, NS B3H 2E2, Canadanunes@dal.ca (A.N.); 2 Faculty of Computer Science, Dalhousie University, Halifax, NS B3K 6R8, Canada

**Keywords:** bipolar disorder, depression, ketamine, lithium, treatment prediction, maintenance therapy

## Abstract

**Background/Objectives:** Lithium remains the first choice for long-term prophylaxis of mood episodes in bipolar disorder (BD), but only 30% of patients will respond, and there is no reliable method by which to predict treatment response. Ketamine is a rapid antidepressant therapy which ostensibly yields greater results in patients with clinical phenotypes that are classically associated with lithium non-response. This inspired a scoping review to map the overlapping and divergent clinical and mechanistic evidence for acute ketamine response and long-term prophylactic lithium therapy in BD. **Methods:** We conducted a scoping review of clinical and preclinical studies that examine convergent and divergent predictors and mechanisms of acute response to ketamine and long-term response to lithium. **Results:** Data from 19 preclinical studies show mechanistic convergence of ketamine and lithium on the GSK-3β/mTOR pathways, and enhancement of synaptic plasticity. Furthermore, lithium appears to consistently limit ketamine-related oxidative stress and hyperlocomotion. However, data from the 23 clinical studies suggest divergence of predictors of response to ketamine and lithium in BD, with ketamine response associated with metabolic risk factors, anxiety/mixed features, and non-melancholic presentations, which are generally predictors of poorer prophylactic lithium response. No study directly tested ketamine response as a predictor of prophylactic lithium response. An important limitation is that clinical studies of ketamine are enriched for lithium-refractory populations and have often included mixed unipolar and bipolar cases. **Conclusions:** Overall, existing data support mechanistic overlap but clinical divergence between ketamine and lithium responders, though this is confounded by sampling bias. We must therefore undertake longitudinal studies of prophylactic lithium therapy among patients with BD who received ketamine for acute antidepressant treatment in order to investigate if responsiveness to ketamine predicts response to lithium, and establish control over BD earlier in the course of illness.

## 1. Introduction

Bipolar disorder (BD) is a chronic neuropsychiatric disorder for which effective long-term maintenance treatment is critical to preventing relapses and reducing morbidity [1]. After more than seven decades, lithium remains the gold-standard prophylactic mood stabilizer in BD [2]. However, only one-third of patients achieve an excellent response, which is characterized by full symptomatic remission and functional recovery [3,4]. Since an unsuccessful lithium trial can entail months of suboptimal treatment and side effects, psychiatrists and researchers must strive to find a priori or early markers of eventual response to lithium prophylaxis [5].

Decades of research suggest that patients with a more “classical” bipolar phenotype are the most likely to respond well to lithium maintenance [4,6,7]. Indeed, lithium responsiveness is associated with a completely episodic course (discrete episodes with full symptomatic remission and functional recovery in the inter-episode period), absence of rapid cycling, a strong family history of BD, and a later age of onset [4,5,8]. By contrast, lithium non-responsive BD patients typically exhibit a more atypical or “non-classical” clinical phenotype [6], including the presence of mixed features, rapid cycling, and significant comorbidities, such as anxiety disorders [4,6,9]. Other studies have noted that metabolic comorbidities, such as insulin resistance, diabetes, and higher body mass index (BMI), are more common in lithium non-responders [8,10]. Since clinicians currently cannot definitively determine lithium response before initiating therapy, many ultimately lithium non-responsive patients are subjected to lengthy lithium trials to determine effectiveness. These patients are at risk of experiencing many months of side-effects and illness recurrence if lithium eventually proves ineffective.

In recent years, ketamine (an NMDA receptor antagonist) has emerged as a novel augmentation option for acute treatment-resistant bipolar depression [11]. Sub-anesthetic doses of ketamine produce rapid antidepressant effects within hours to days, which compares favorably to the 4–8 weeks typically required for response to conventional antidepressants [12,13]. Intriguingly, ketamine appears to benefit some subgroups of patients that are traditionally difficult to treat, such as patients with non-melancholic or “anxious” depressive subtypes [14], or patients with significant comorbid mixed mood features [15,16]. Furthermore, a higher body mass index (BMI) has predicted positive response to ketamine [17]. Taken together, these findings suggest that acute ketamine responders may phenotypically resemble prophylactic lithium non-responders, implying that ketamine’s mechanism of action may potentially target acute depressive pathology in patient populations whose clinical phenotypes diverge from those typically observed in responders to long-term lithium prophylaxis.

Therefore, we sought to understand whether non-response to an acute ketamine trial could serve as a positive predictor of long-term lithium efficacy, and whether a robust response to ketamine might predict a lower probability of lithium maintenance success through the evaluation of both clinical and preclinical evidence. The implications of such a relationship, if proven, would be highly significant. The rapid antidepressant effect of ketamine offers a unique opportunity to biologically “test” a patient’s treatment responsiveness within days, whereas evaluating lithium’s prophylactic effect requires months to years of observation. If a brief ketamine infusion series could reliably indicate whether a patient is more or less likely to respond to prophylactic lithium, the efficiency of long-term treatment decisions in BD could be greatly improved.

Up front, an important methodological consideration arises from the inclusion criteria of most clinical trials of ketamine in bipolar depression; specifically, that studying treatment-resistant samples will increase the probability that included patients had already failed to respond to multiple prior pharmacologic interventions, including mood stabilizers such as lithium. As a result, these study populations are likely enriched for individuals who are already known or presumed to be lithium non-responders. This could create a systematic sampling bias limiting the generalizability of findings and obscuring potential relationships between ketamine and lithium response. Thus, we considered it important to examine the overlapping and distinct mechanisms of action of ketamine and lithium in order to evaluate their common and differential effects in patients with BD. This paper therefore presents a scoping review of the clinical and preclinical evidence supporting or refuting this proposed link between ketamine response and lithium maintenance outcomes in BD.

## 2. Methods

**Figure 1:**
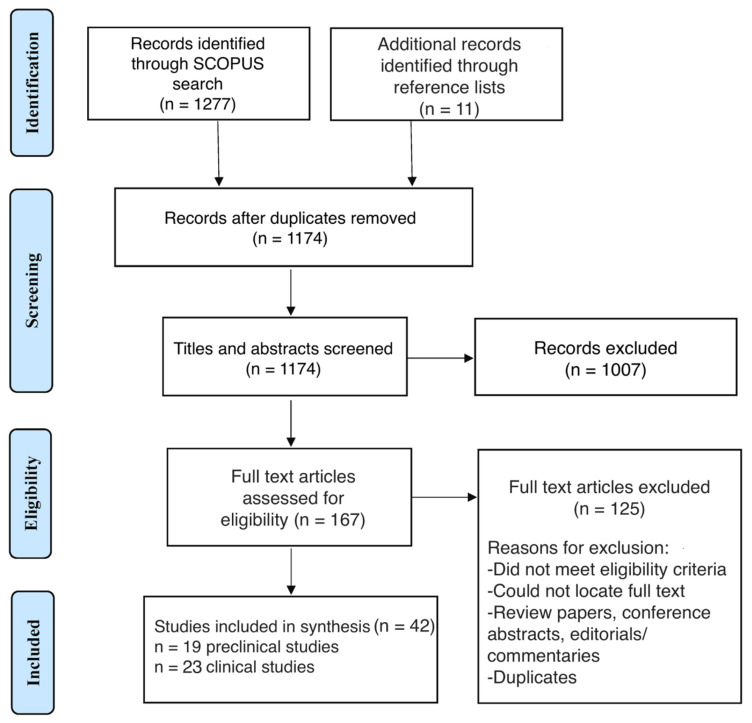
Preferred Reporting Items for Systematic Reviews and Meta-Analyses (PRISMA) flow diagram of study selection.

This scoping review adheres to the Preferred Reporting Items for Systematic Review and Meta-Analysis for Scoping Reviews (PRISMA-ScR) guidelines (Figure 1) [18].

### 2.1. Data Collection

The Scopus database was searched from its inception to 26 April 2025, using the following search query:

TITLE-ABS-KEY (ketamine AND (lithium OR anticonvuls* OR antipsychot* OR mood-stabiliz* OR (mood PRE/0 stabiliz*)) AND (bipolar OR depress*)). Reviews, conference abstracts, commentaries, and editorials were excluded from the review but their reference lists were manually examined to identify additional potentially relevant publications for inclusion.

### 2.2. Screening Process

Two authors (J.T. and A.N.) independently assessed the titles and abstracts of the retrieved articles for potential inclusion. Disagreements between reviewers were resolved through follow-up discussion to reach a consensus. Inter-rater reliability was not formally assessed during screening. One author (J.T.) then assessed full-text papers published in English for final inclusion. Articles were included in the present review if they met the following criteria: (1) Clinical trials that investigated the use of ketamine and a mood stabilizer in the treatment of bipolar depression, and which reported depressive symptom severity ratings over time; (2) Studies that administered ketamine in combination with a mood stabilizer to animals; (3) Preclinical translational studies that explored the mechanistic relationship between ketamine and mood stabilizers, examining both overlapping and distinct mechanisms; (4) Studies that specifically evaluated clinical predictors of ketamine response. Unpublished data, reviews, commentaries, and editorial articles were not eligible for inclusion in the present review. We included studies where ketamine was administered with other prophylactic treatments, such as lamotrigine and clozapine, in order to further explore the mechanistic overlap and divergence between ketamine, lithium, and these other prophylactic agents.

For all studies, data extracted by J.T. and A.N. included study information (e.g., author, publication details), participant/animal population, study design, intervention details, and relevant depressive outcomes. Study data were then categorized, summarized, and qualitatively synthesized by the study team. Despite the limitations of preclinical studies in capturing the complexity of BD, relatively equal weighting was given to the data extracted from preclinical and clinical studies, as the mechanistic insights gained from preclinical evidence helped address our study aims.

## 3. Results

The initial search yielded a total of 1277 records, with 11 additional records identified from relevant reference lists (Figure 1). After removing 114 duplicates, 1174 records were screened for relevance based on titles and abstracts, with 1007 excluded at this stage. Following full-text review of 167 articles, 125 studies were excluded based on the eligibility criteria previously outlined. In total, 42 records that met the inclusion criteria were included in the qualitative synthesis, 19 of which were preclinical studies and 23 of which were clinical studies.

### 3.1. Preclinical Studies

#### 3.1.1. Lithium Augmentation of the Rapid Antidepressant Effect of Ketamine

Chiu et al. [19] showed that a single administration of high-dose ketamine (50 mg/kg delivered intraperitoneally [i.p.]) reverses depressive-like behavior in chronically stressed mice (measured as a reduction in immobility in forced swim and tail suspension tests). However, this antidepressant effect is transient and accompanied by increased oxidative stress markers in the brain. Notably, low-dose lithium given before ketamine enabled a previously ineffective subthreshold ketamine dose (2.5 mg/kg) to produce antidepressant-like behavioral effects. Likewise, lithium delivered post-ketamine (at therapeutic levels) sustained ketamine’s antidepressant action and synaptic enhancements for at least 2 weeks. Furthermore, Chiu et al. found that lithium pretreatment completely abolished ketamine-induced oxidative damage (lipid peroxidation, glutathione oxidation) in the stressed mouse brain [19]. Mechanistically, lithium-enhanced ketamine-mediated activation of the mTOR–BDNF pathway in the prefrontal cortex (PFC) resulted in increased dendritic spine density associated with antidepressant response. This suggests that combining ketamine with lithium can both potentiate the acute antidepressant effect and limit ketamine-related adverse oxidative stress.

Ketamine’s antidepressant action may be related to GSK-3β inhibition and consequent increases in synaptic plasticity. Liu et al. [20] demonstrated that blocking GSK-3, either with lithium or a selective inhibitor, markedly potentiated ketamine’s effects. In their study, a normally subthreshold ketamine dose produced robust antidepressant-like effects only when combined with a lithium/GSK-3 inhibitor, resulting in rapid mTORC1 activation, elevated BDNF–TrkB signaling, and increased density/strength of synaptic spines. The combination (low-dose ketamine + lithium) was as effective as a higher ketamine dose alone, highlighting GSK-3 as a mediator of ketamine’s synaptogenic and behavioral effects. As a clinical corollary, this may also support a treatment strategy for patients who may not be able to tolerate normally therapeutic ketamine doses (i.e., potentiation with a low dose of lithium). In line with this, do Vale et al. [21] found that lithium synergized with ketamine to enhance antidepressant outcomes and anti-inflammatory signaling. Low-dose ketamine (2 mg/kg) had only modest effects on forced-swim immobility, but when paired with lithium it yielded a reduction in immobility time comparable to a 10 mg/kg ketamine dose. This combination also potentiated a ketamine-mediated reduction in pro-inflammatory markers (TNF-α, iNOS, COX-2) and increased inhibitory GSK-3β phosphorylation in peripheral tissue. These findings suggest that GSK-3 inhibition is a critical mechanism that boosts ketamine-driven antidepressant response and synaptic plasticity.

Recent data also implicate insulin–Akt (insulin-linked kinases) signaling in ketamine’s sustained antidepressant effect. In a rodent model of treatment-resistant depression, Price et al. [22] showed that adding lithium to ketamine not only reduced immobility time and increased latency to immobility in the forced swim test, but also uniquely upregulated insulin pathway signaling. Rats receiving ketamine and lithium had significantly higher plasma insulin levels and mTOR activation, along with increased phosphorylation of Akt in the prefrontal cortex. Blood insulin levels correlated inversely with depression-like immobility in these animals receiving both ketamine and lithium, and increased insulin signaling in the infralimbic PFC also correlated with behavioral improvement. These results suggest lithium’s augmentation may recruit metabolic signaling pathways to support antidepressant effects.

At the circuit level, ketamine’s antidepressant action appears to be related to its acute psychotomimetic effects. For example, Maltbie et al. [23] used fMRI in awake nonhuman primates to map ketamine-induced brain activation. Ketamine infusion broadly increased BOLD activation, with the strongest activations in the cingulate gyrus, supplementary motor area (SMA), and thalamus, but this was reduced in both extent and magnitude by risperidone pretreatment. There was no deactivation of the subgenual cingulate observed—an area where overactivity has been implicated in treatment-resistant depression [24]. It is thus possible that disinhibition of PFC networks is a shared mechanism for the rapid antidepressant effect of ketamine and its transient psychotic symptoms. The lack of acute antipsychotic effects of lithium in humans may thus suggest that divergence in the effects of ketamine and lithium could relate to mechanisms related to psychotomimesis.

Further circuit-related findings suggest that ketamine and lithium may overlap in their effects on stress-related brain circuits. Specifically, in a study of rats by Stepan et al. [25] using hippocampal slice imaging, the authors found that chronic stress impairs propagation of neural activity through the hippocampus, whereas lithium, ketamine, and BDNF ameliorate this impairment. Such findings suggest that ketamine and lithium both reinforce synaptic connectivity in stress-sensitive circuits, further supporting convergent mechanistic targets for these agents.

#### 3.1.2. Ketamine as a Preclinical Model of Mania/Psychosis

Repeated low-dose ketamine in rodents can produce hyperactivity and dysregulation thought to be analogous to manic episodes. This ketamine-induced “mania-like” behavior is typically characterized by increased locomotor activity, risk-taking, and dysregulated neurotransmitter signaling. A consistent finding across such studies is that ketamine triggers oxidative stress and inflammation in brain regions involved in mood regulation (prefrontal cortex, hippocampus, striatum), and therefore many interventions that prevent ketamine’s “mania-like” side-effects are thought to have potent antioxidant or neuroprotective properties.

For example, a series of studies from Gazal and colleagues explored dietary antioxidants as prophylactics in a ketamine mania model. In multiple studies, pre-treatment with lithium or a blueberry extract markedly protected rats against ketamine-induced hyperlocomotive and oxidative/inflammatory effects in the cortex, striatum, and hippocampus [26,27]. Additionally, pre-treatment with an extract of blackberry (another anthocyanin-rich berry) attenuated ketamine-induced hyperlocomotion and brain oxidative stress markers in a manner similar to lithium [28]. Pretreatment with curcumin [29] (the active component of turmeric), an extract of *Cecropia pachystachya* [30], or gallic acid [31], has also been shown to limit ketamine’s hyperlocomotive effects and oxidative/inflammatory markers, particularly in the cortex, hippocampus, and striatum, to a degree similar to pretreatment with lithium. Gallic acid was also able to normalize acetylcholinesterase activity in the hippocampus and striatum, countering ketamine’s increase in acetylcholinesterase activity, as was lithium [31]. However, in another study, the free-radical ederavone, unlike lithium, failed to prevent hyperlocomotion in the open-field test [32]. Taken together, these findings suggest that lithium (and other antioxidants) limit ketamine’s hyperlocomotive effects (which, in this model, represent mania), and may attenuate the oxidative/inflammatory responses of ketamine administration.

Beyond oxidative/inflammatory pathways, lithium may limit ketamine-induced hyperlocomotion through other mechanisms. For example, lithium has been shown to limit widespread ketamine-induced forebrain activation in limbic regions, such as the hippocampus, amygdala, lateral septum, and hypothalamus [33], suggesting that lithium can dampen the aberrant circuit excitability underlying ketamine’s “mania-like” effect, potentially explaining lithium’s clinical anti-manic efficacy. To some degree, lithium-related attenuation of ketamine-induced hyperlocomotion may be related to lithium downregulating PI3K–Akt signaling in the medial PFC [34]. That same study found that pharmacological inhibition of mTOR (with rapamycin) had no effect on hyperlocomotion [34]. It is therefore possible that lithium counteracts ketamine’s hyperlocomotive effects, not only via the antioxidant mechanisms mentioned in the previous section, but also via the dampening of neural excitability, potentially via activity in the PI3K–Akt pathway.

To better understand the mechanistic convergence and divergence between ketamine and lithium, it may also be helpful to examine the mechanisms of alternative mood stabilizers and antipsychotics when co-administered with ketamine. For example, lamotrigine has been found to prevent ketamine-induced deficits in prepulse inhibition (PPI), which is an operational measure of sensorimotor gating that is reduced in mania and schizophrenia [35]. Interestingly, in that study, lamotrigine did not reduce the amphetamine-induced PPI deficit, implying that lamotrigine specifically antagonizes glutamate/NMDA-related gating disturbance (i.e., ketamine’s PPI effect) but not dopamine-driven PPI deficit (i.e., due to amphetamine). Clinically, this may align with lamotrigine’s efficacy in bipolar maintenance, supporting a glutamatergic modulation role in normalizing circuit function. On the other hand, the atypical antipsychotic clozapine may reverse hippocampal-prefrontal impairments in long-term potentiation (LTP) induced by the administration of ketamine [36]. While both ketamine and clozapine increased phospho-GSK3β in the PFC in that study, clozapine uniquely increased GluA1 phosphorylation in the PFC and hippocampus (enhancing AMPA channel conductance at those sites). This pattern indicates an interesting overlap between ketamine, clozapine, and lithium with respect to the effects on GSK3β, suggesting that clozapine and lamotrigine’s effects on countering ketamine-related mania/psychosis may be mediated by the effects on glutamatergic transmission.

Using EEG, Bowman et al. [37] used freely moving rats to examine how clozapine and another modulator, naltrexone, influenced ketamine-induced network oscillations, with the clinically important implication being that ketamine’s EEG signature of psychosis vs. antidepressant response might be separable. Ketamine alone produced distinct resting-state EEG abnormalities, including a reduction in low-beta power and an increase in gamma- and high-frequency oscillations during resting states (identified via head-mounted accelerometers). Clozapine reversed the low-beta deficit, which may be related to its antipsychotic action. Clozapine also potentiated ketamine’s high-frequency oscillation increases, whereas naltrexone (which in human patients blocks ketamine’s antidepressant and anti-suicidal effects) [38] specifically blunted the high-frequency oscillation component, which may thus be a marker of ketamine’s antidepressant action.

Taken together, these preclinical studies suggest lithium is effective at augmenting the antidepressant effect of ketamine while also proving effective at curtailing ketamine’s inducing of mania-like states. This creates a complex preclinical picture, leaving the relationship between acute ketamine response and long-term lithium prophylaxis an open clinical question.

### 3.2. Clinical Studies

#### 3.2.1. Rapid Antidepressant Efficacy of Ketamine in Mood Disorders

Ketamine has demonstrated significant antidepressant effects in both treatment-resistant bipolar and unipolar depression. In the first randomized placebo-controlled trial in bipolar depression, a single sub-anesthetic IV dose of ketamine (0.5 mg/kg) administered to patients receiving stable levels of lithium or valproate produced significantly improved depressive symptoms within 40 min, with 71% of the 17 patients responding at two weeks (compared to only 6% of the 16 patients in the placebo arm) [39]. There was no treatment response stratification by lithium or valproate treatment status. A replication study confirmed these findings [40], and overall these add-on trials (with patients maintained on lithium or valproate throughout) indicate that ketamine’s efficacy is compatible with concurrent mood stabilizer therapy. Furthermore, no relationship has been found between blood levels of lithium or valproate and the antidepressant response to ketamine [41] (although this study may have been underpowered). An open-label clinical series in 53 patients with bipolar depression on stable doses of mood stabilizers further corroborated the concurrent effectiveness of ketamine, with no clear stratification of response across bipolar subtypes and illness histories, number of prior episodes, or concomitant lithium/quetiapine use [42]. Still another study found that lithium or anticonvulsant use was unrelated to response trajectories in patients with severe depression (in a mixed sample of unipolar and bipolar depression) [43]. Taken together, these studies suggest that the acute antidepressant effects of ketamine are not affected by mood stabilizer use.

#### 3.2.2. Symptom Subtypes and Clinical Predictors of Response

Interestingly, ketamine’s antidepressant efficacy may differ depending on the depression subtype. In an observational study of 97 patients with treatment-resistant depression, Wang et al. [14] found that those with melancholic features were significantly less likely to respond or to achieve remission, and improved more slowly compared to patients with anxious or non-melancholic depression following six ketamine infusions. These findings suggest that patients with melancholic features might require additional measures or may respond less robustly to ketamine’s antidepressant mechanism, whereas those with significant anxious distress (at least in the absence of melancholic signs) could be particularly good candidates for ketamine therapy. While there is difficulty in clinical settings in distinguishing anxious distress from mixed features in depression [44], this finding remains of particular interest given that prior studies have suggested that melancholic features may predict favorable outcomes with lithium prophylaxis [45], indicating further clinical divergence between predictors of lithium and ketamine response. Similarly, in bipolar depression, anxiety comorbidity does not appear to diminish ketamine’s efficacy. A post hoc analysis focusing on bipolar patients with high baseline anxiety found that patients with anxious bipolar depression (*n* = 21) derived a similar magnitude and timing of benefit from ketamine as those with non-anxious (*n* = 15) bipolar depression [15]. This efficacy of ketamine in anxious bipolar depression indicates it may bypass some of the limitations of conventional treatments in these populations.

Beyond mood subtype, other clinical factors like personality and trauma history have emerged as potential moderators of ketamine response. A recent study of intranasal ketamine (esketamine) in depressed inpatients reported that patients with a co-morbid personality disorder (the most common of which was borderline personality disorder) had a significantly lower response rate to ketamine, whereas patients with a history of childhood trauma had a higher likelihood of responding [46]. Although this study was exploratory in nature and prone to the risk of uncorrected multiple comparisons, and inclusive of a mixed unipolar/bipolar sample, it poses an interesting further potential contrast between the clinical predictors of response to ketamine and prophylactic lithium therapy, as histories of childhood trauma are predictive of a poorer prophylactic response to lithium [47]. Given that there is some evidence that childhood maltreatment may not decrease responsiveness to anticonvulsant mood stabilizers, it is of potential interest to consider whether acute ketamine antidepressant response may be differentially predictive of response to prophylactic anticonvulsant therapy [48].

An analysis by Pennybaker et al. [49] examined which clinical features distinguished longer-lasting (what they called “extended”) antidepressant responses to ketamine, finding that extended responders were both more likely to have a family history of alcohol use disorder in first-degree relatives and to experience greater acute dissociation during the ketamine infusion [49]. Another study found that ketamine responders may have a higher intra-infusion increase in systolic blood pressure compared to non-responders, although the effect on the duration of response was not reported [50]. No clinical predictors that are known to influence prophylactic lithium response in bipolar disorder were identified. Thus, it remains unclear whether the duration of ketamine’s antidepressant response provides any information about the likelihood of a subsequent prophylactic lithium response. There has also been mixed evidence of clinical laboratory measures, such as vitamin B12 and VEGF predicting response to ketamine [51,52], with no known overlap with predictors of lithium response.

#### 3.2.3. Adjunctive Treatments to Sustain Ketamine’s Effects

Because ketamine’s antidepressant benefit is often transient, adjunctive strategies to prolong its effect have been tested. To date, the only randomized controlled trial investigating lithium continuation following ketamine response in patients with treatment-resistant unipolar depression [53] found that lithium offered no advantage over the placebo in maintaining antidepressant response. This suggests that lithium alone, started immediately after ketamine, does not reliably prevent early relapse. Notably, the mean plasma level of lithium in the study was 0.61 mEq/L, toward the lower end of target ranges for both the acute treatment of bipolar depression and the maintenance treatment typically recommended in the guidelines [1]. By contrast, in a 5-year observational follow-up study in 16 patients, Amiaz et al. [54] found that patients receiving quetiapine experienced a longer time until relapse compared to patients receiving other neuroleptics. Patients (a mixed sample of unipolar and bipolar disorder) who received ketamine while on quetiapine had a median time to relapse of ~966 days compared to ~80 days for those on other antipsychotics (aripiprazole, olanzapine, ziprasidone, perphenazine, and clozapine).

#### 3.2.4. Neuroimaging and Neurophysiological Mechanisms

Neuroimaging studies have suggested that ketamine’s antidepressant response in BD may be related to the rapid reorganization of brain networks. In a PET imaging study of depressed bipolar patients who did not respond to 4-week trials of either lithium or valproate, and who subsequently underwent a double-blind crossover trial of ketamine vs. saline placebo, Nugent et al. [55] showed that antidepressant responders exhibited the greatest post-infusion increases in ventral striatal metabolism, while higher activity in the subgenual anterior cingulate cortex (sgACC) following the placebo infusion predicted greater symptom relief following ketamine. Along these lines, resting-state fMRI findings in patients with MDD have found that ketamine responders showed lower baseline functional connectivity between the lateral prefrontal cortex and the sgACC, as well as larger increases following treatment. Indeed, Chen et al. [56] also found that patients with treatment-resistant depression showed lower fronto-striatal functional connectivity, and that the magnitude of this connectivity was inversely related to the degree of antidepressant improvement following IV ketamine [56]. Furthermore, in a repeated-infusion protocol, patients with unipolar depression who responded to IV ketamine demonstrated elevated baseline resting-state functional connectivity between various regions and the amygdala [57]. These differences may be related to oscillatory patterns of activity in depression, since Cao et al. [58] reported that responders to IV ketamine showed lower frontal power in the theta band at baseline, but higher treatment-related increases in alpha power, as well as lower alpha asymmetry and theta cordance. The degree to which these findings are associated with a prophylactic response to lithium is unclear, as we are unaware of evidence associating functional connectivity and EEG-biomarkers with prophylactic mood stabilizer response.

#### 3.2.5. Peripheral Biomarkers and Molecular Pathways

One striking finding involves ketamine’s effect on the glutamate–mTOR signaling pathway, which is involved in synaptic plasticity, and may be a significant mediator of ketamine’s antidepressant action. In a small study of 27 depressed women, Berner et al. [59] found that higher baseline levels of phosphorylated p70S6 kinase (p70S6K, a downstream effector of mTORC1 whose phosphorylation levels served as a proxy for MTORC1 activation in monocytes) were correlated with better antidepressant outcomes from ketamine, although the study used a machine learning method that in such a small sample would be prone to overfitting. Notwithstanding this limitation, the implication here may be that individuals with higher baseline MTORC1 activity may be potentially more “primed” to respond to the antidepressant effects of ketamine, which involves synaptogenesis and may depend on mTOR-mediated pathways. mTOR may also serve as a convergence point between the acute antidepressant effects of ketamine and the longer-term prophylactic effects of lithium in bipolar disorder, since a key mechanism of the latter involves GSK3β inhibition, an upstream regulator of mTOR activity.

In a placebo-controlled clinical study, a single ketamine infusion significantly increased vascular endothelial growth factor A (*VEGFA*) gene expression in whole blood of depressed patients compared to a midazolam (sedative) control [60]. Ketamine also selectively increased the ratio of *VEGFA* to its anti-angiogenic counterpart *PEDF* (pigment epithelial-derived factor, with gene name *SERPINF1*) in peripheral blood, measured about 4 h post-infusion [60]. The specificity of these biomarker changes in the ketamine infusion group may suggest that ketamine engages pro-angiogenic growth factor signaling. Interestingly, in rats, it has been demonstrated that lithium promotes VEGF expression in brain endothelium and astrocytes [61].

In a study by Permoda-Osip et al. [52] of 42 depressed patients, personal/family history of alcohol abuse, elevated serum vitamin B12 concentrations, and elevated serum vascular endothelial growth factor predicted response to ketamine. Villaseñor et al. [62] found that lysophosphatidylethanolamines and lysophosphatidylcholines were increased in ketamine responders relative to non-responders in a sample of patients with bipolar depression, suggesting that mitochondrial fatty acid metabolism may predict response to ketamine. Słupski et al. [63] showed that repeated ketamine infusions decreased serum copper concentrations in depressed patients, though no clear connection to treatment response was found. Reduced copper concentrations may be due to a reduction in the acute phase response, further suggesting ketamine exerts anti-inflammatory effects.

## 4. Discussion

This review examined the relationship between the acute antidepressant effects of ketamine and the long-term prophylactic outcomes with lithium in bipolar disorder (BD). We found that, while ketamine and lithium share convergent molecular targets that enhance structural plasticity, their clinical predictors of efficacy largely diverge. In this section, we synthesize this evidence, attempt to consider why such divergence may exist, and discuss the implications for clinical prediction and the design of future studies leveraging ketamine response to predict prophylactic response to lithium.

Ketamine and lithium act on overlapping molecular pathways implicated in neuroplasticity. Ketamine produces rapid antidepressant effects through NMDA receptor antagonism, resulting in glutamate surges and AMPA receptor activation, and ultimately downstream mTORC1–BDNF signaling, which together promote rapid synaptogenesis [64,65]. Over a longer time scale, lithium exerts pro-synaptic effects via GSK-3β inhibition, disinhibiting mTOR and supporting neurotrophic and gene-expression changes that sustain long-term mood stabilization [19,66]. Rodent studies have suggested that these mechanisms may compound each other’s effects. Indeed, lithium potentiates and prolongs the antidepressant effects of subthreshold ketamine doses, and reduces ketamine-induced oxidative stress [19,20,21]. Both agents upregulate phosphorylation of GSK-3β and mTOR in the prefrontal cortex, reinforcing structural plasticity [19]. As a result, lithium attenuates ketamine-induced hyperlocomotion and limbic hyperactivation, potentially consistent with its anti-manic effects in humans [33,34]. These findings support a shared capacity to enhance synaptic strength and, especially for lithium, protect against excitotoxicity, suggesting some mechanistic overlap among biological responders. In terms of clinical overlap in function, it is notable that both agents reduce suicidal ideation, albeit over different time scales (ketamine within hours [40,67] and lithium across years of maintenance [68]). A corollary question that arises from this overlap therefore concerns the degree to which synaptic plasticity is involved in the pathophysiology of suicidal ideation and behavior.

Despite the aforementioned clinical overlaps, the clinical phenotypes of ketamine and lithium responders differ significantly. Excellent lithium responders typically present with “classical” BD, characterized by episodic euphoric manias, discrete illness episodes, complete inter-episode remission, later onset, and strong family history [69,70]. This stands in stark contrast to the atypical clinical phenotypes that are ostensibly characteristic of ketamine responders and lithium non-response. Indeed, while high body mass index (BMI) predicts ketamine response [17,71], factors such as insulin resistance and metabolic syndrome are associated with lithium non-response [10,72]. Similarly, while comorbid anxiety and mixed features limit lithium prophylaxis [4,6,9], they are associated with positive ketamine response [15,16]. Childhood trauma predicts poor lithium outcomes [47] but may enhance ketamine responsiveness [46]. Finally, depression subtypes diverge: melancholic features favor lithium [45], whereas non-melancholic or anxious depressions respond preferentially to ketamine [14]. Taken together, these findings suggest that ketamine responders are overrepresented with respect to the features that portend worse prognosis for longer-term prophylactic lithium responsiveness.

Ultimately, our review failed to identify clear preclinical evidence explaining the divergent phenotypic profiles of responders to acute ketamine treatment and long-term lithium prophylaxis. Animal models reliably show that ketamine produces rapid acute antidepressant effects which can be extended or modulated by lithium, but do not provide clinically relevant approaches to identifying whether and which ketamine responders are less likely to benefit from lithium prophylaxis. For example, while oxidative stress and inflammatory changes accompany ketamine’s behavioral effects in rodents [26,27,28], these findings do not straightforwardly map onto the clinical observation that ketamine responders often show the phenotypic predictors of lithium non-responders. Similarly, while insulin and metabolic pathways are implicated in both ketamine’s and lithium’s actions [22], animal studies do not yet clarify why metabolic dysregulation (e.g., high BMI) would favor acute ketamine response but undermine lithium prophylaxis [10].

One possible explanation for our failure to reconcile the preclinical and clinical data is that preclinical models largely capture the acute effects of ketamine and lithium, such as how lithium extends the duration of the antidepressant effect of ketamine or counteracts ketamine-induced hyperlocomotion. By contrast, the clinical divergence in response emerges over years of illness course (since lithium responsiveness requires more than 1–2 years to elucidate), reflecting potential differences in the mechanisms of long-term disease stabilization versus short-term symptom relief. Unfortunately, animal models are not well suited to capturing these long-horizon prophylactic outcomes. This limits our ability to test hypotheses about divergent predictors of response at the biological level. Thus, while the clinical data suggest an inverse predictive relationship between ketamine response and prophylactic lithium response, the preclinical literature remains largely neutral on this matter.

In summary, convergent mechanisms of action between ketamine and lithium in BD include action on GSK-3β/mTOR pathways as well as the enhancement of synaptic plasticity. Ketamine-induced oxidative stress and hyperlocomotion are consistently limited by lithium. Clinical predictors of response seem to diverge; ketamine response is associated with metabolic risk factors, anxiety/mixed features, and non-melancholic presentations, which are generally predictors of prophylactic lithium non-response. Taken together, this suggests the potential for a personalized medicine approach, whereby response to ketamine in patients with bipolar depression could serve as a biomarker of non-response to lithium (pending prospective validation), saving individuals prolonged ineffective trials of lithium prophylaxis.

Several limitations must be acknowledged. No clinical study has directly tested ketamine response as a predictor of lithium prophylaxis, so all inferences remain indirect. Additionally, many of the clinical studies included herein included mixed unipolar/bipolar samples, which were not disentangled in post hoc analyses. Third, and arguably most significantly, ketamine trials are likely enriched for treatment-resistant patients, implying that they may contain a higher proportion of lithium non-responders, which may inflate the appearance of divergence. That is, due to ketamine typically being reserved for treatment-resistant populations, many patients receiving ketamine are a priori lithium non-responders due to previously failed lithium trials. As prior medication trials were scarcely defined in the clinical studies used within this evidence synthesis, it is unclear how many of the study populations examined were enriched for lithium non-responders. Additionally, this temporal relationship of medication usage is incongruent with the clinical question initially outlined—that is, is responsiveness to ketamine predictive of responsiveness to lithium. What may appear to be a divergence in the clinical phenotype of responders to ketamine vs. lithium may be an artifact of sampling bias, whereby samples enriched for lithium non-responsiveness who respond to ketamine (or any other treatment) will appear to diverge from classical lithium responders with respect to clinical, neurophysiological, and biochemical factors. Furthermore, many predictor studies, both for ketamine and lithium, are small, underpowered, and heterogeneous in design, complicating interpretation. Finally, one must also note that preclinical studies, while mechanistically valuable, cannot model the full complexity of BD or the longitudinal trajectories that are required to understand prophylactic lithium response, limiting their utility in understanding this critical element of disease management in humans.

## 5. Conclusions

The existing literature supports mechanistic overlap but clinical divergence between ketamine and lithium responders. No studies directly examined ketamine response as a predictor of lithium nonresponse, and using existing data to investigate this hypothesis comes with many limitations. Most notably, ketamine trials are enriched for treatment-resistant populations, and clinical divergence between ketamine and lithium responders may simply be reflective of ketamine responders being a priori lithium non-responders. To resolve these uncertainties, prospective, longitudinal studies must be conducted in which individuals with BD who have received acute courses of ketamine for depression are followed prospectively while receiving lithium prophylaxis. Incorporating biomarkers such as inflammatory and metabolic measures, neuroimaging, and electrophysiology may help link clinical observations to mechanistic pathways, and induced pluripotent stem cell-based assays may help qualify overlapping and diverging mechanistic pathways at the individual level [73,74,75,76,77]. Preclinical work should additionally focus on developing animal models of the longitudinal relapsing and remitting trajectory of BD, to better understand prophylaxis. Ultimately, such studies would serve to determine whether ketamine response, which could be a rapid and easily identified biomarker, predicts response to lithium prophylaxis, thereby potentially saving patients years of ineffective trials of prophylactic therapy. If validated in prospective cohorts, such a precision medicine approach could help establish control over the disease earlier, improving both morbidity and mortality in this patient population.

## Data Availability

No new data were created or analyzed in this study. Data sharing is not applicable.
